# Photo-Activated Psoralen Binds the ErbB2 Catalytic Kinase Domain, Blocking ErbB2 Signaling and Triggering Tumor Cell Apoptosis

**DOI:** 10.1371/journal.pone.0088983

**Published:** 2014-02-14

**Authors:** Wenle Xia, David Gooden, Leihua Liu, Sumin Zhao, Erik J. Soderblom, Eric J. Toone, Wayne F. Beyer, Harold Walder, Neil L. Spector

**Affiliations:** 1 Department of Medicine, Duke University Medical Center, Durham, North Carolina, United States of America; 2 Duke Cancer Institute, Duke University Medical Center, Durham, North Carolina, United States of America; 3 Department of Chemistry, Research Drive, Duke University, Durham, North Carolina, United States of America; 4 QNS Group, LLC, Bahama, North Carolina, United States of America; 5 Immunolight LLC, Detroit, Michigan, United States of America; H. Lee Moffitt Cancer Center & Research Institute, United States of America

## Abstract

Photo-activation of psoralen with UVA irradiation, referred to as PUVA, is used in the treatment of proliferative skin disorders. The anti-proliferative effects of PUVA have been largely attributed to psoralen intercalation of DNA, which upon UV treatment, triggers the formation of interstrand DNA crosslinks (ICL) that inhibit transcription and DNA replication. Here, we show that PUVA exerts antitumor effects in models of human breast cancer that overexpress the ErbB2 receptor tyrosine kinase oncogene, through a new mechanism. Independent of ICL formation, the antitumor effects of PUVA in ErbB2+ breast cancer models can instead be mediated through inhibition of ErbB2 activation and signaling. Using a mass spectroscopy-based approach, we show for the first time that photo-activated 8MOP (8-methoxypsoralen) interacts with the ErbB2 catalytic autokinase domain. Furthermore, PUVA can reverse therapeutic resistance to lapatinib and other ErbB2 targeted therapies, including resistance mediated via expression of a phosphorylated, truncated form of ErbB2 (p85^ErbB2^) that is preferentially expressed in tumor cell nuclei. Current ErbB2 targeted therapies, small molecule kinase inhibitors or antibodies, do not block the phosphorylated, activated state of p85^ErbB2^. Here we show that PUVA reduced p85^ErbB2^ phosphorylation leading to tumor cell apoptosis. Thus, in addition to its effects on DNA and the formation of ICL, PUVA represents a novel ErbB2 targeted therapy for the treatment of ErbB2+ breast cancers, including those that have developed resistance to other ErbB2 targeted therapies.

## Introduction

8-Methoxypsoralen (8MOP) is a linear tricyclic molecule that readily enters cell nuclei where it intercalates DNA at pyrimidine-purine sites [1]. Following photo-activation by UV irradiation, a combination referred to as PUVA, 8MOP interacts with pyrimidines to form stable DNA monoadducts. Upon further UVA treatment, a percentage of monoadducts can then be converted to interstrand DNA crosslinks (ICL), which in turn inhibit transcription and DNA replication [1], [2]. Importantly, the anti-proliferative effects of PUVA appear to be related to the formation of ICL, rather than monoadducts. Because of its anti-proliferative effects, PUVA has been used to treat hyperproliferative skin conditions including psoriasis [3]. Furthermore, T lymphocytes- normal and malignant- appear to be particularly sensitive to the anti-proliferative effects of PUVA therapy; hence, the use of PUVA as a treatment for cutaneous T-cell lymphoma and graft-versus-host disease [4]–[6].

In addition to playing a role in the formation of ICL, there is evidence that psoralen may also target non-nuclear proteins, lipids, and cellular membrane components [7]–[9]. For example, Laskin *et al* used psoralen derivatives incapable of forming DNA adducts in response to UV irradiation to show that PUVA treatment blocked the mitogenic effects of soluble Epidermal Growth Factor (EGF) on its cognate cell surface receptor, EGF Receptor (EGFR) [7], [9]. Interestingly, inhibition of EGFR phosphorylation in response to PUVA was not mediated through a direct psoralen-EGFR interaction, but rather psoralen interacting with a lower molecular weight binding protein.

Similar to EGFR, the ErbB2 oncogene is a member of the type 1 transmembrane family of receptor tyrosine kinases. Gene amplification and overexpression of ErbB2, which occurs in 25% of all breast cancers, predicts for a poor clinical outcome as a consequence of increased tendency to metastasize to visceral organs earlier in the disease course [10], [11]. These findings have prompted the development of ErbB2 targeted therapies- biological and small molecule tyrosine kinase inhibitors (TKIs)- for the treatment of early and advanced stage ErbB2+ breast cancers [12]. Although ErbB2 targeted therapies represent a significant advancement in the treatment of aggressive breast cancers, their clinical efficacy has been limited by the inevitable development of therapeutic resistance, particularly in the advanced stage setting [13]–[15].

Using mass spectroscopy and biochemical approaches, we now show for the first time that photo-activated 8MOP can directly interact with regulatory elements within the ErbB2 catalytic kinase domain, providing a likely explanation for the targeted inhibition of ErbB2 signaling in response to PUVA therapy. Furthermore, a modified psoralen derivative that lacks the ability to crosslink DNA maintained its ability to block ErbB2 signaling and induce tumor cell apoptosis. Importantly, we show that PUVA can trigger significant apoptosis in ErbB2+ breast cancer models of acquired therapeutic resistance to lapatinib and similar ErbB2 targeted therapies. These findings and their clinical implications will be further discussed.

## Materials and Methods

### Cell Culture and Reagents

ErbB2+ (BT474; SKBR3) and ErbB2 negative (MCF-7; T47D) human breast cancer cell lines, and the human foreskin fibroblast (HFF) cell line were obtained from the American Type Culture Collection (Manassas, VA). Lapatinib resistant breast cancer cells (rBT474; rSKBR3) were generated and maintained in culture as previously described [16]–[18]. Cells were maintained in RPMI-1640 supplemented with 10% fetal bovine serum and L-glutamine from GIBCO (Grand Island, NY) growing in a humidified atmosphere of 5% CO_2_ at 37°C. IRDye 800 conjugated affinity purified anti-rabbit IgG and anti-mouse IgG were from Rockland (Gilbertsville, PA). Alexa Fluor 680 goat anti-rabbit IgG were purchased from Molecular Probes (Eugene, OR). Anti-PARP (Poly (ADP-ribose) Polymerase) monoclonal antibody was from BD PharMingen (San Jose, CA). 8-Methoxypsoralen (8MOP) and the 4G10 anti-phosphotyrosine (p-tyr) antibody were purchased from Sigma-Aldrich (St. Louis, MO). Monoclonal antibodies to c-ErbB2 and EGFR were purchased from Neo Markers (Union City, CA). PARP cleavage product was obtained from Cell Signaling (Beverly, MA). Antibodies to Akt1/2, phospho-Akt1/2 (S473), phospho-Akt1/2 (T308), phospho-Erk1/2 and Erk1/2 were purchased from Santa Cruz (Santa Cruz, CA). Lapatinib (GW572016) and neratinib (HKI-272) [19] were purchased from LC Laboratories (Woburn, CA).

### UV Irradiation, Growth/Viability and Apoptosis Assays

UV irradiation was carried out in 6 or 96 well plate format in a UV Stratalinker 1800 (Statagene, LA Jolla, CA) at the UV doses indicated in the figures. Cell growth and viability assays were performed in a 96-well plate format in a final volume of 100 µl/well using the cell proliferation reagent WST-1 from Roche Diagnostics (Mannheim, Germany), as previously described [16]–[18]. Details of the apoptosis assay have been previously described [16]–[18]. Briefly, cells were treated in 12-well plates with 8MOP, UV irradiation, or lapatinib at the treatment conditions indicated in the Figure legends. Cells were harvested with trypsin-EDTA, and 5000 cells in final volume of 50 µl were sampled in 96-well microplates. Cells were directly stained with annexin V-PE and nexin 7-AAD in 1× Nexin Buffer in a 200 µl final reaction volume. After incubating at room temperature for 20 min, the reaction samples were analyzed in the Guava PCA-96-system (Guava Technology Inc. Hayward, CA).

### SDS-PAGE and Western Blot Analysis

Whole cell extracts were prepared by scraping cells off petri dishes, washing cell pellets 2x in phosphate buffered saline (PBS), and then re-suspending pellets in two-packed-cell volumes of RIPA buffer (150 mM NaCl, 50 mM Tris-HCl, pH 7.5, 0.25% (w/v) deoxycholate, 1% NP-40, 5 mM sodium orthovanadate, 2 mM sodium fluoride, and a protease inhibitor cocktail). Protein concentrations were determined using a modification of the Bradford method (Bio-Rad Labs, Hercules, CA). For Western blot analysis, equal amounts of proteins (25 to 50 µg) were resolved by either 7.5% or 4–15% gradient SDS polyacrylamide gel electrophoresis under reducing conditions as previously described [16]–[18]. Proteins were transferred to nitrocellulose membranes and probed with primary antibodies specific for proteins of interest. After extensive washing, membranes were incubated with a secondary IRDye 800 conjugated anti-rabbit or mouse IgG, or Alexa Fluor 680 anti-rabbit IgG and proteins were visualized using the LI-COR Odyssey Infrared Imaging System (LI-COR, Inc., Lincoln, NE).

### Protein Pull-down and Nano-Flow Liquid Chromatography Electrospray Ionization Tandem Mass Spectrometry (LC-MS/MS) Analysis

BT474 cells were pre-treated with 5 µM biotin-linked 8-MOP for 24 hr before being irradiated with 1J UVA. After UVA irradiation, cells were harvested and whole cell lysates were prepared in RIPA buffer. After centrifugation, a 30 µl suspension of M-280 Streptavidin Dynabeads® (Invitrogen, Carlsbad, CA) was added to each 130 µl crude lysate sample. The resulting mixtures were placed on an orbital vortex mixer for 20–30 min. The samples were then magnetized and the supernatants removed and discarded. A solution of 0.01% (v/v) Tween 20 in PBS (150 µl) was added to each sample and the resulting mixtures were placed on orbital vortex mixer for 20–30 min. The samples were then magnetized and the supernatants were removed and discarded. Next a solution of 0.1% (v/v) SDS in PBS (150 µl) was added to each sample and the resulting mixtures were heated to 50°C for 15 min. The samples were magnetized and supernatants removed and discarded, after which 150 µl of 50 mM ammonium bicarbonate was added to each sample, and the mixtures placed on orbital vortex mixer for 20–30 min. The samples were again magnetized and the supernatants removed and discarded. The samples were suspended in 130 µl ammonium bicarbonate (50 mM) prior to mass spectroscopy analysis. Following a pull-down of biotinylated-drug on immobilized streptavidin magnetic beads, samples were washed three times with 200 µl 50 mM ammonium bicarbonate, pH 8. Sample volume was brought to 100 µl 50 mM ammonium bicarbonate (pH 8), and supplemented with Rapigest surfactant (Waters Corporation, Milford, MA) to a final concentration of 0.1%. Following disulfide reduction with 5 mM dithiolthreitol at 40°C for 20 min, free sulfhydryls were alkylated with 10 mM iodoacetamide at room temperature for 45 min. Approximately, 500 ng of sequencing grade modified trypsin (Promega Corporation, Madison, WI) was added to each sample and on-resin digestion was allowed to occur for 18 hr at 37°C with orbital shaking. Supernatants were then collected from each sample after centrifugation at 1000 g for 2 min and Rapigest surfactant was hydrolyzed by acidification to 0.5% trifluoracetic acid (final pH 2.5) for 2 hr at 60°C. Following desalted by C18 Zip-Tip (Millipore) SPE, samples were brought to dryness by vacuum centrifugation and finally resuspended in 10 µl 2% acetonitrile, 0.1% formic acid. Peptide mixtures were subjected to chromatographic separation on a Waters NanoAcquity UPLC (New Objective, Cambridge, MA) equipped with a 1.7 µm BEH130 C_18_ 75 µm I.D. ×250 mm reversed-phase column. The mobile phase consisted of (A) 0.1% formic acid in water and (B) 0.1% formic acid in acetonitrile. Following a 5 µl injection, peptides were trapped for 5 min on a 5 µm Symmetry C_18_ 180 µm I.D. ×20 mm column at 20 µl/min in 99.9% (A). The analytical column was then switched in-line and a linear elution gradient of 5% B to 40% B was performed over 90 min at 300 nL/min. The analytical column was connected to a fused silica PicoTip emitter (New Objective, Cambridge, MA) with a 10 µm tip orifice and coupled to a Waters QToF Premier mass spectrometer through an electrospray interface. The instrument was operated in a data-dependent mode of acquisition with the top three most abundant ions selected for MS/MS using a charge state dependent CID energy setting with a 60 s dynamic exclusion list employed. Mass spectra were processed with Mascot Distiller (Matrix Science) and were then submitted to Mascot searches (Matrix Science) against a SwissProt_human database appended with reverse entries at 20 ppm precursor and 0.04 Da product ion mass tolerances with trypsin protease rules selected. Dynamic mass modifications corresponding to oxidation on Met residues were allowed. Searched spectra were imported into Scaffold v2.5 (Proteome Software) and scoring thresholds were set to yield a protein false discovery rate of 0.2% (implemented by the PeptideProphet algorithm) based on decoy database searches.

### Gene Transfection of p85^ErbB2^in Human Breast Cancer Cells

The c-terminal fragment (p85^ErbB2^) was generated based on ErbB2 open reading frames and sub-cloned into the pcDNA 3.1 (+) as previously described [17]. HER2 negative T47D breast cancer cells were transfected with the p85 expressing vector using the Lipofectamine™ 2000 Reagent from Invitrogen (Carlsbad, CA) as previously described [17]. Stably transfected cells were selected using G418 (400 µg/ml) and the expression level of p85^ErbB2^ was confirmed by western blot analysis as previously described [17].

### Statistical Analysis

Data were expressed as means with standard error bars included. Student's *t*-test was used to determine statistical significance between 2 groups. A value of p<0.05 was considered a statistically significant difference.

## Results

### Inhibition of ErbB2 signaling triggers apoptosis in PUVA-treated ErbB2+ breast cancer cells

**Figure 1:**
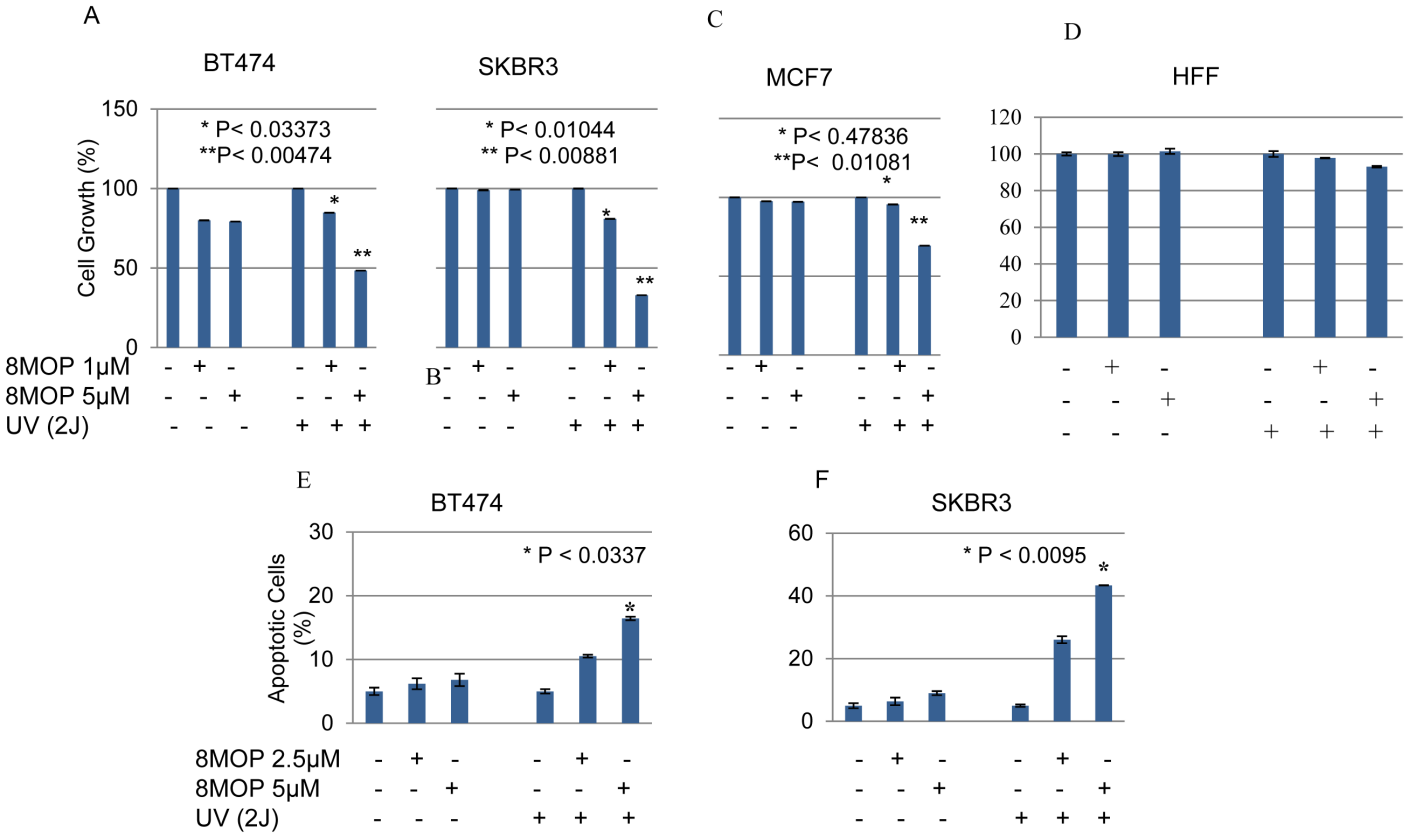
PUVA antitumor activity in HER2 + breast cancer cells. Cells were pre-treated with the indicated concentrations of 8MOP for 4 hr before UVA irradiation (2J), and then cultured for an additional 72 hr before being analyzed for cell growth (A) BT474; (B) SKBR3; (C) MCF7, (D) HFF and apoptosis (D) BT474; (E) SKBR3. Cells treated with vehicle (0.01% DMSO) alone served as controls. Results represent the mean +/− standard error of triplicate samples, and are representative of three independent experiments.

**Figure 2:**
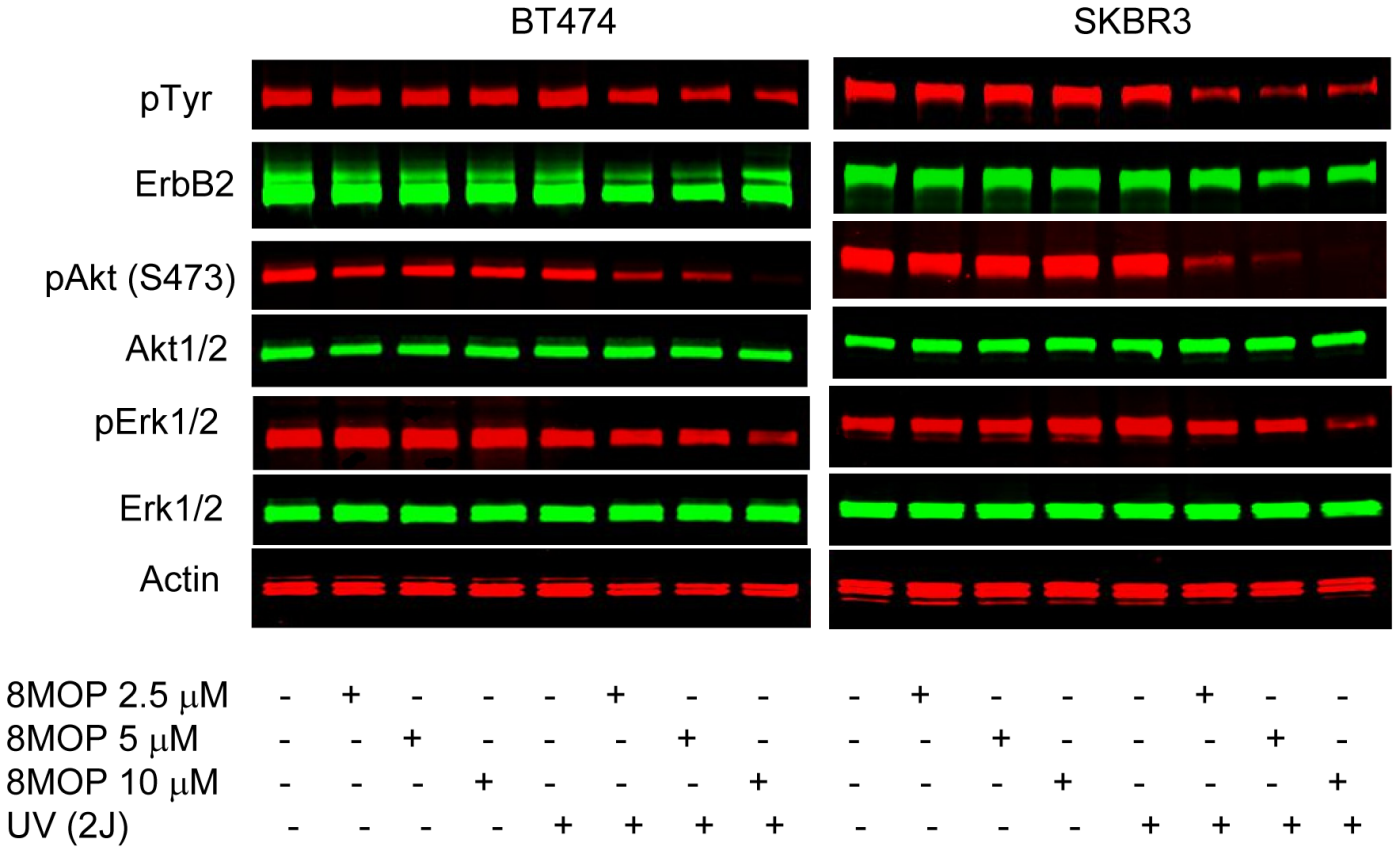
PUVA therapy inhibits ErbB2 signaling. BT474, SKBR3 and MCF7 cells were subjected to the indicated treatment conditions as described in Figure 1. Western blot analysis was performed on whole cell lysates. Actin steady-state protein levels served as a control to ensure for equal loading of protein. Results are representative of three independent experiments.

The growth and viability of ErbB2+ breast cancer cell lines was significantly inhibited by PUVA therapy in a dose-dependent manner (Figure 1A and B). The loss of tumor cell viability appeared to be related to induction of apoptosis (Figure 1E and F). In contrast, PUVA therapy using identical treatment conditions (2.5 and 5 µM 8MOP) had relatively less effect on the growth and viability of MCF7 cells, a ErbB2 non-overexpressing human breast cancer cell line and a non-malignant human foreskin fibroblast cell line (HFF) (Figure 1C and D). It was therefore tempting to speculate that photo-activated 8MOP might directly modulate ErbB2 activation and signaling. Seeking to demonstrate the effects of PUVA on ErbB2 signaling, we showed that steady-state protein levels of the activated, phosphorylated form of ErbB2 were reduced in PUVA-treated ErbB2+ breast cancer cell lines in a dose-dependent manner (Figure 2). In addition, total ErbB2 protein levels were reduced in response to higher doses of PUVA therapy. In addition, the activated, phosphorylated forms of Akt and Erk1/2, which are key downstream mediators of the PI3K and MAPK signaling pathways, respectively were also inhibited by PUVA (Figure 2). In contrast, treatment with the same dose of UV irradiation or 8MOP alone had relatively little effect on cell viability or ErbB2 signaling (Figures 1 and 2).

### Psoralen can directly interact with the ErbB2 catalytic kinase domain

**Figure 3:**
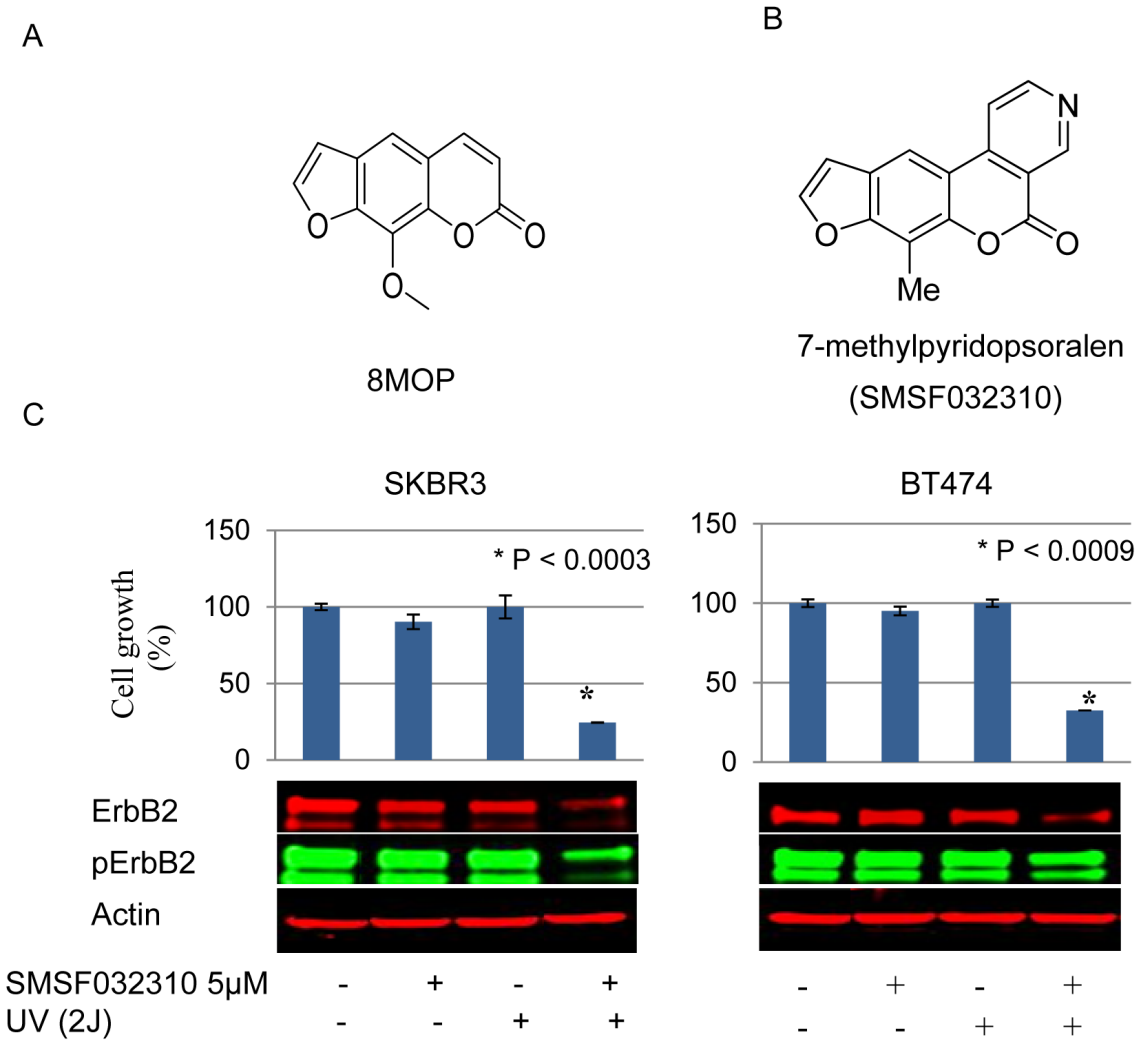
Inhibition of ErbB2 signaling in response to PUVA is independent of DNA crosslinking. The chemical structures of (A) 8MOP, and (B) 7-methylpyridopsoralen (SMSF032310), which is a derivative of 8MOP that lacks the ability to crosslink DNA, are shown. (C) BT474 and SKBR3 cells were exposed to the indicated treatments. Cell growth and viability assays were performed after 72 hr. *P*<0.0003 (SKBR3); *P*<0.0009 (BT474). Cells treated with vehicle alone served as controls. Results represent the mean +/− standard error of triplicate samples, and are representative of three independent experiments. Corresponding Western blot analysis of the indicated protein/phosphoproteins is shown. Steady-state actin protein levels served as controls for equal loading of proteins. Results are representative of three independent experiments.

**Figure 4:**
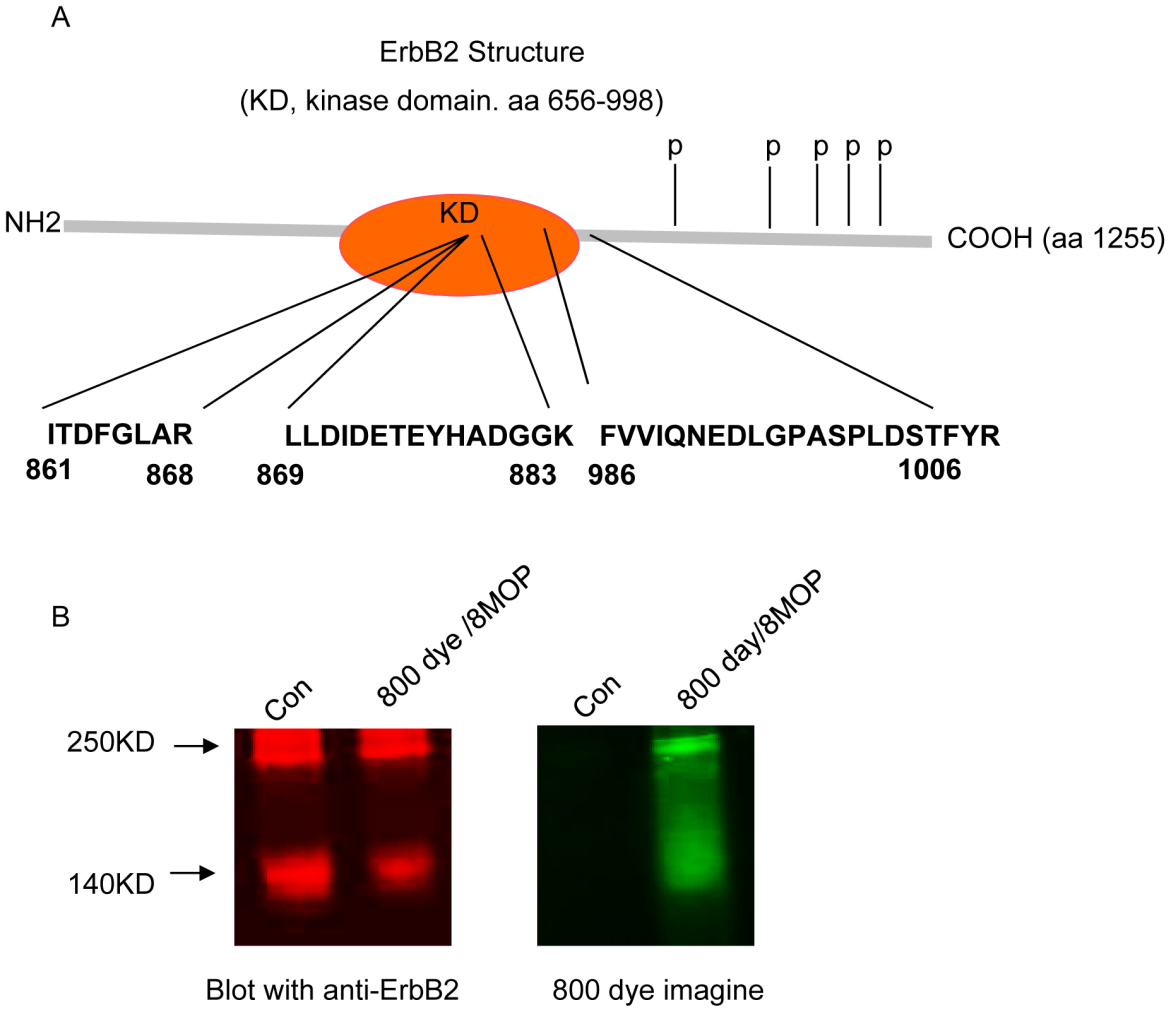
8MOP interacts with the catalytic kinase domain of ErbB2. (A) 8MOP interacts with three peptide regions within the ErbB2 catalytic kinase domain. Qualitative peptide identifications within the ErbB2 catalytic kinase domain following LC-MS/MS analysis of a streptavidin pull-down of biotinylated-8MOP bait (see Materials and Methods). The transmembrane domain is indicated (red diamond) and the five C-terminus tyrosine autophosphorylation sites are indicated (p). (B) Non-reducing Western blot analysis of the interaction of 8MOP with ErbB2. BT474 cells were treated with 800dye-8MOP (Promega) or with vehicle (0.01% DMSO) alone served as control for 48 hr and then exposed to UV irradiation (2J) prior to Western blot analysis. The image on the left shows the Western blot for ErbB2 (red). The image on the right shows the same membrane directly scanned for the presence of 800dye-8MOP (green), which overlays the ErbB2 signal. The results are representative of three independent experiments.

We synthesized a derivative of psoralen, 7-methylpyridopsoralen (Figure 3A), which lacks the DNA binding motif, making it unable to generate ICLs. We next sought to determine the effects of this compound on ErbB2 signaling and tumor cell viability following UVA irradiation. Upon photo-activation, 7-methylpyridopsoralen significantly inhibited the growth and viability of ErbB2+ breast cancer cells, which correlated with inhibition of phosphorylated and total ErbB2 protein expression (Figure 3). These findings suggested that interruption of ErbB2 signaling in PUVA-treated tumor cells can be mediated by a mechanism(s) independent of ICL formation. We next sought to determine whether 8MOP can directly interact with the catalytic kinase domain of ErbB2. In this regard, BT474 cells were treated with biotinylated-8MOP and a pull-down experiment was performed (see Materials and Methods). Biotinylated-8MOP-protein complexes were isolated from BT474 cell lysate on immobilized streptavidin magnetic beads and subjected to protein digestion using sequencing grade modified trypsin. Peptides were then isolated by LC-MS/MS (see Materials and Methods). We identified three 8MOP bound peptides that corresponded to two sites located within the catalytic kinase domain (aa 861-868; aa 869-883), and one site in the peptide crossover kinase domain (aa 986–1006) (Figure 4A). As a second independent approach to demonstrate the interaction between 8MOP and ErbB2, BT474 cells were treated with fluorophore-labeled 8MOP (see Materials and Methods) and UVA irradiation. Cell lysates were separated under non-denaturing conditions using native gel electrophoresis. Proteins were transferred to a PVDF membrane, and the fluorophore-labeled 8MOP detected by an Odyssey scanner (Figure 4B, green). The membrane was then blotted with a primary fluoro-labeled ErbB2 antibody (Figure 4B, red). The fluoro-conjugated 8MOP was detected at the same molecular weight as large ErbB2 complexes, findings that were consistent with the LC-MS/MS data indicating that 8MOP can directly interact with the ErbB2 receptor.

### PUVA plus neratinib leads to enhanced tumor cell killing

**Figure 5:**
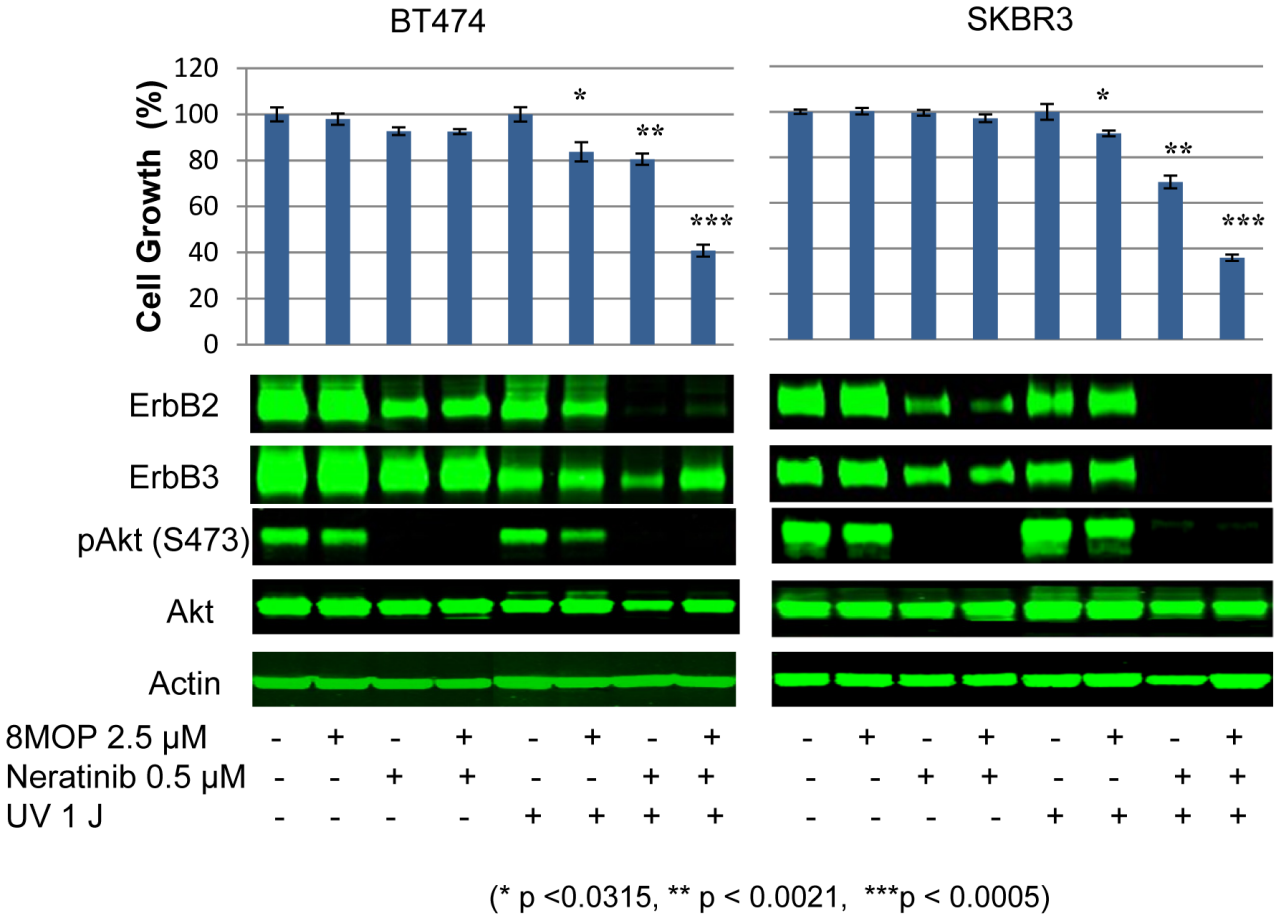
The combination of PUVA with the irreversible pan-ErbB TKI neratinib results in enhanced antitumor activity. The growth and viability of BT474 and SKBR3 cells (top bar graphs) after being subjected to the indicated treatment conditions. The combination of PUVA plus neratinib: *P*<0.0005 (BT474 and SKBR3 cells). Results represent the mean +/− standard error of triplicate samples, and are representative of three independent experiments. (B) Western blot analysis showing steady-state ErbB2, ErbB3, and phospho-Akt (S473) protein levels in BT474 and SKBR3 cells treated according to the indicated treatment conditions. Vehicle alone (0.01% DMSO) served as a control. Steady-state actin protein levels served as a control for equal loading of protein. The results are representative of three independent experiments.

Targeted therapies tend to be more clinically efficacious in combination with other targeted or cytotoxic drugs. We therefore evaluated a variety of targeted agents in combination with PUVA including PI3K inhibitors, HDAC inhibitors, PARP inhibitors, and other ErbB TKIs. The most promising combination was with neratinib (HKI-272), a small molecule, irreversible pan-ErbB (ErbB1/EGFR; ErbB2; ErbB3; ErbB4) tyrosine kinase inhibitor that is currently in late phase clinical trials [19]. Treatment of ErbB2+ breast cancer cells with the combination of 8-MOP, UVA irradiation, and neratinib, each at sub-lethal doses when used alone, resulted in significantly enhanced inhibition of cell viability (Figure 5). The effects of this combination on ErbB2, ErbB3 and downstream signaling pathways were further analyzed. Consistent with our recent findings [16], we found that neratinib treatment alone resulted in a marked reduction in total ErbB2 and ErbB3 protein levels, with consequential loss of p-Akt (S473) expression. Interestingly, neratinib in combination with UVA irradiation alone led to further loss of total ErbB2 and ErbB3 protein expression, which was more pronounced in SKBR3 cells (Figure 5).

### PUVA treatment can reverse lapatinib resistance in HER2+ breast cancer cells

**Figure 6:**
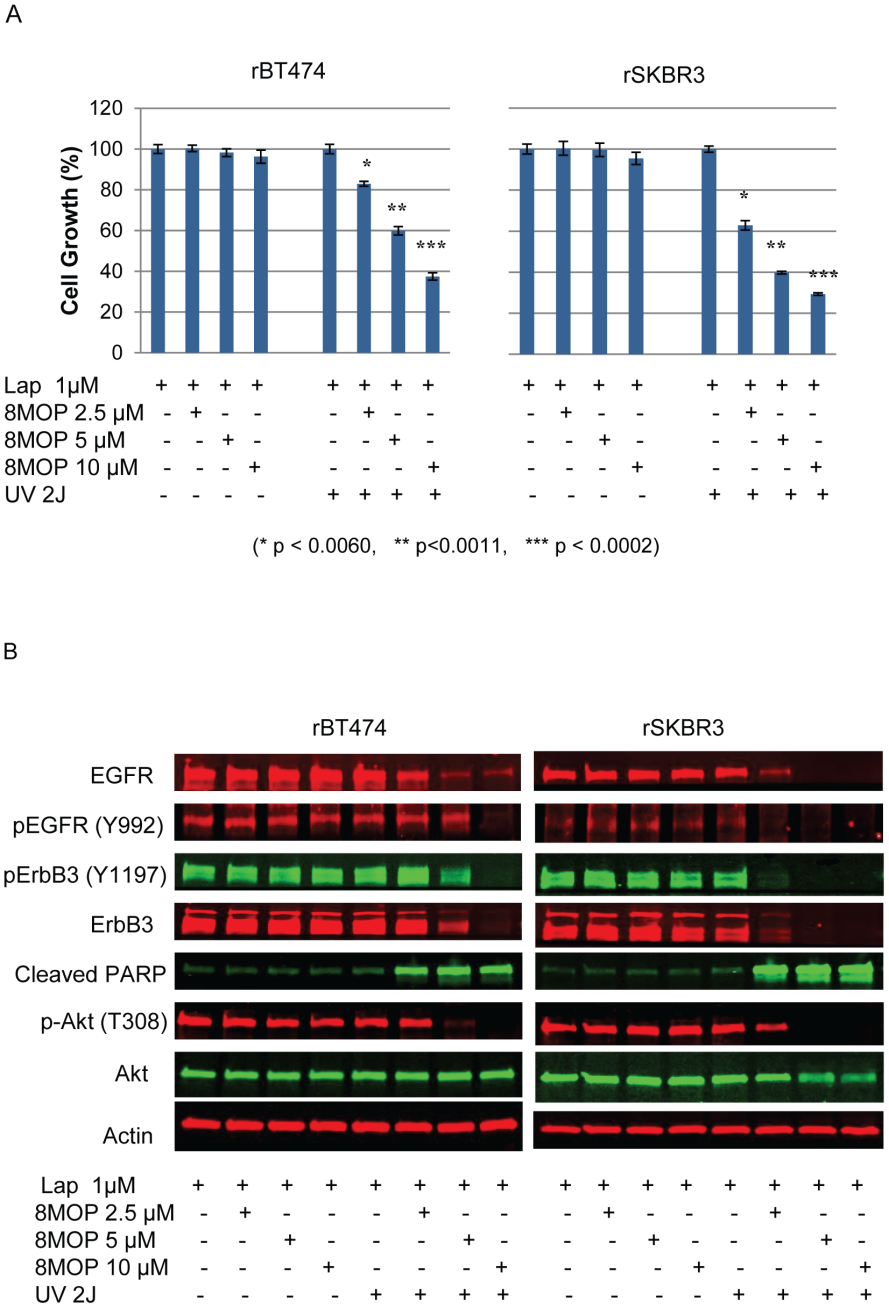
PUVA therapy reverses acquired resistant to ErbB2 targeted therapies. (A) Equal numbers of rBT474 and rSKBR3 cells were subjected to the indicated treatment conditions, and the effects on cell growth and viability are shown. *P* values of statistical significance are indicated. Results represent the mean +/− standard error of triplicate samples, and are representative of three independent experiments. (B) The corresponding Western blot analysis for each of the indicated treatment conditions is shown. As indicated, rBT474 and rSKBR3 cells are continuously maintained in 1 µM lapatinib. Actin steady-state protein levels served as a control to ensure for equal loading of protein. Results are representative of three independent experiments.

We recently showed that development of acquired therapeutic resistance to the reversible HER2 and EGFR tyrosine kinase inhibitor lapatinib in HER2+ breast cancer cells can be mediated by a number of mechanisms including: (i) a switch in the regulation of cell survival from HER2-HER3-PI3K signaling in treatment naïve cells to EGFR-HER3-PI3K in resistant cells [16]; and (ii) expression of a truncated ErbB2 form preferentially expressed in tumor cell nuclei [17]. We next sought to determine whether PUVA treatment could reverse lapatinib resistance. Using models of lapatinib resistance established in our laboratory [16]-[18], we treated rBT474 and rSKBR3 cells with PUVA at the indicated concentrations of 8MOP (Figure 6A). Resistant cells were continuously maintained in the presence of 1 µM lapatinib. As shown, PUVA treatment significantly reduced tumor cell growth and viability in a dose-dependent manner (Figure 6A). In contrast to isotype-matched parental cells (Figure 5), total EGFR and ErbB3 protein expression was markedly reduced in PUVA-treated rBT474 and rSKBR3, in addition to reduction in the expression of phosphorylated forms of EGFR (Y992), ErbB3 (Y1197), and Akt (T308) (Figure 6B). The effects of PUVA on Akt T308 are particularly interesting in light of our recent finding that Akt T308, but not S473, remained persistently phosphorylated in lapatinib resistant cells [16]. Importantly, PARP cleavage product was increased in PUVA-treated lapatinib resistant tumor cells consistent with induction of apoptosis.

**Figure 7:**
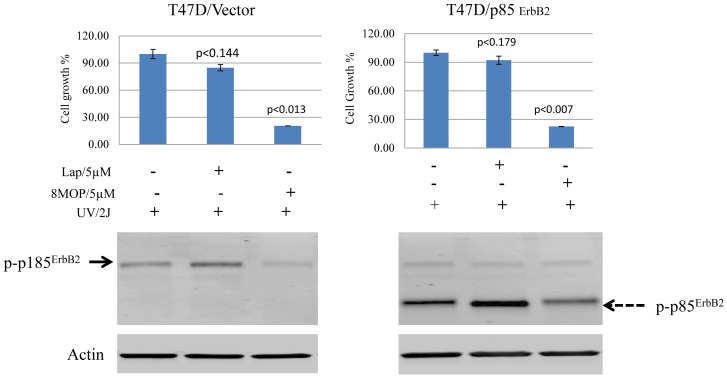
PUVA therapy targets nuclear p85^ErbB2^, inducing tumor cell apoptosis. Top bar graph shows the results of the growth assays performed in T47D and stably transfected T47D cell line. T47D cells expressing p85^ErbB2^ were pretreated with 5 µM lapatinib or 5 µM 8MOP for 4 hr followed by irradiation in a UV Stratalinker 1800 (Statagene). Cells transfected with empty vector (T47D/Vector), and those treated with vehicle alone (0.01% DMSO) served as controls. The effects of the treatments on cell growth and viability are shown in the bar graph. P<0.0071 (8MOP + UVA irradiation). Results represent the mean +/− standard error of triplicate samples, and are representative of three independent experiments. Steady-state phospho-p85^ErbB2^ protein levels (dotted arrow) and phospho-p185^ErbB2^ (solid arrow) are shown by Western blot. Actin steady-state protein levels served as a control to ensure for equal loading of protein. Results are representative of three independent experiments.

We have also shown that expression of an 85 kDa truncated form of ErbB2 (p85^ErbB2^) that lacks the extracellular and transmembrane domains, is preferentially expressed in the nuclei of tumors that have become resistant to lapatinib [17]. Moreover, expression of p85^ErbB2^ under the control of a heterologous promoter can render cells resistant to lapatinib and other ErbB2 targeted drugs in otherwise sensitive ErbB2+ breast cancer cells [17]. Although p85^ErbB2^ is tyrosine phosphorylation, an indication of its activated state, it is not inhibited by lapatinib (Figure 7) [17]. To study the effects of PUVA on p85^ErbB2^, we established a T47D transfected breast cancer cell that stably expresses phosphorylated p85^ErbB2^ in tumor cell nuclei as previously described [17]. T47D cells, although not HER2 overexpressing, still express full-length HER2 (Figure 7). PUVA therapy has an antitumor effect in T47D cells transfected with empty vector alone that is associated full-length ErbB2 (p185^ErbB2^). However, in p85 expressing T47D cells, treatment with PUVA, but not lapatinib, markedly inhibited p85^ErbB2^ phosphorylation, triggering tumor cell apoptosis (Figure 7).

## Discussion

The molecular basis for the anti-proliferative effects of photo-activated psoralen in the treatment of benign and neoplastic skin diseases has historically been attributed to the formation of interstrand DNA crosslinks that lead to inhibition of transcription and DNA replication. T cells, which mediate many of the dermatological indications for PUVA e.g. graft-versus-host disease; cutaneous T cell lymphoma seem to be particularly sensitive to the anti-proliferative effects of PUVA and have therefore served as a frequently used model to study the biological effects of PUVA therapy [4]-[6]. In contrast, there has been relatively little scientific evidence to support the use of PUVA therapy in the treatment of solid tumors. Here, we show for the first time that PUVA therapy can directly target the catalytic kinase domain of the ErbB2 receptor tyrosine kinase oncogene. The interaction of photo-activated 8MOP with regulatory elements within the ErbB2 catalytic kinase domain may explain the marked inhibition of ErbB2 signaling in PUVA-treated ErbB2+ breast cancer cells, including those that have developed resistant to current FDA-approved ErbB2 targeted therapies.

The interaction between 8MOP and ErbB2 was demonstrated using two independent strategies: (i) LC/MS/MS; and (ii) Western blot analysis. These findings were further supported by the observation that a DNA non-crosslinking psoralen derivative maintained its ability to block ErbB2 signaling and induce tumor cell apoptosis. The ErbB2+ breast cancer cell lines used in these studies express EGFR, which has also been shown to be a target of PUVA therapy [9]. However, survival of parental BT474 and SKBR3 cells is not dependent upon EGFR, but instead, dependent upon signaling via ErbB2-ErbB3 heterodimers [16]. Our data suggests that the antitumor effects of PUVA therapy in parental ErbB2+ breast cancer cells were mediated through direct effects on ErbB2. Moreover, non-malignant HFF cells that express wild-type EGFR were less sensitive to the apoptotic effects of PUVA, consistent with the notion that EGFR is not responsible for induction of apoptosis in PUVA-treated ErbB2+ breast cancer cells (Figure 1D).

Of particular interest is the antitumor activity of PUVA therapy in ErbB2+ breast cancer models of acquired therapeutic resistance to lapatinib and other ErbB2 targeted therapies. It is worth noting that acquired therapeutic resistance to lapatinib does not appear to be mediated by reactivation of ErbB2 signaling. In fact, ErbB2 phosphorylation remains inhibited in resistant cells [16], [18]. Importantly, targeted molecular knockdown of ErbB2 does not reverse lapatinib resistance [18], indicating that survival of resistant cells is no longer dependent upon ErbB2 alone, at least not the 185 kDa full-length form of ErbB2 (p185^ErbB2^) expressed at the cell surface. Instead, the viability of lapatinib resistant ErbB2+ breast cancer cells is dependent upon other factors. For example, we have shown that resistant cells express a truncated form of ErbB2, referred to as p85^ErbB2^, which can be generated by alternate initiation of translation [20] and/or proteolytic processing of p185^ErbB2^ [17]. Moreover, p85^ErbB2^ is preferentially expressed in tumor cell nuclei. This nuclear, truncated form of ErbB2 lacks the extracellular (ECD) and transmembrane domains, while retaining the full cytoplasmic domain, including the catalytic kinase domain and tyrosine autophosphorylation sites. Expression of p85^ErbB2^ driven by a heterologous promoter renders ErbB2+ breast cancer cells that are normally sensitive to the antitumor effects of lapatinib, resistant to lapatinib and other ErbB2 targeted therapies. Although the exact mechanism of p85^ErbB2^ action is unknown, it, in contrast to p185^ErbB2^ and p110^ErbB2^- a membrane-bound form of ErbB2 that lacks the ECD- does not appear to activate cytoplasmic protein kinase signaling cascades [17]. We have shown that tyrosine phosphorylation of p85^ErbB2^ is not inhibited by lapatinib or similar TKIs in class. We now show that PUVA therapy blocks p85^ErbB2^ phosphorylation, triggering apoptosis. One potential explanation is that lapatinib cannot access the ATP binding groove of p85^ErbB2^. In contrast, the ability of 8MOP to access nuclear targets e.g. DNA, is well-established. It is therefore tempting to speculate that 8MOP more readily accesses, and blocks the catalytic kinase domain of p85^ErbB2^.

We recently showed that development of lapatinib resistance can be mediated through a switch in the regulation of cell survival from ErbB2-ErbB3-PI3K signaling in treatment naïve ErbB2+ breast cancer cells to ErbB3-EGFR-PI3K-PDK1-Akt (T308) signaling axis in the resistant setting, the latter driven in part through autocrine production of the ErbB3 ligand heregulin β1[16]. Although the exact mechanism(s) underlying the antitumor effects of PUVA is unknown, persistent phosphorylation of Akt T308, which was seen in models of lapatinib resistance [16], was inhibited by PUVA. In addition, total EGFR and ErbB3 were reduced in PUVA-treated resistant cells. These findings are interesting in light of a recent study showing that several ErbB TKIs can induce proteolysis of targeted receptor(s) in a manner similar to hsp90 antagonists [21]. Induction of receptor proteolysis by TKIs, including lapatinib, was shown to be mediated through ATP competitive binding with cdc37, the latter stimulating binding between the client protein e.g. ErbB2 and hsp90. It is therefore tempting to speculate that PUVA therapy may also trigger degradation of EGFR, ErbB2, and ErbB3 by blocking access of the cdc37/hsp90 complex to client proteins ErbB receptors.

We propose that 8-MOP interacts with the ErbB2 catalytic kinase domain at amino acid residues distinct from lapatinib. Although structural analysis of a lapatinib-EGFR complex has been reported, there has been no structural analysis of a lapatinib-ErbB2 complex. Most of the amino acid residues associated with the regulation of the ErbB2 catalytic kinase activity are located in the vicinity of the ATP binding groove within the deep cleft located between the N- and C-terminal lobes of the ErbB2 receptor [22]. We found that 8MOP interacts with peptides located within the DFG motif and activation loop of the C-lobe, both of which are involved in regulating ErbB2 autokinase activity [22]. The structural analysis of lapatinib-EGFR crystals suggests that lapatinib likely interacts with amino acid residues within the ATP binding groove distinct from those of photo-activated 8MOP [22]. It is possible therefore that photo-activated 8-MOP binds to the catalytic kinase domain, blocking its activity and triggering proteolysis of the receptor in a manner similar to irreversible ErbB TKIs e.g. neratinib.

Targeted therapies are increasingly being used in combination with other targeted and cytotoxic drugs [23]. We were interested to find out whether other targeted therapies might enhance the antitumor activity of PUVA therapy. In this regard, a recent study found that the combination of a histone deacetylase (HDAC) inhibitor and PUVA led to enhanced antitumor activity compared with either treatment alone [24]. When we examined the effects of adding targeted therapies, including HDAC and PI3K inhibitors, to PUVA therapy, we found that neratinib, at sub-lethal doses alone, significantly increased apoptosis in ErbB2+ breast cancer cells when combined with PUVA. It is known that neratinib has promiscuous inhibitory activity against non-ErbB kinases, including MAP kinase family members. It is possible that the enhanced antitumor effect observed with the addition of neratinib to PUVA might be directly or indirectly related to inhibition of a kinase(s) involved in DNA repair of ICL, thereby sensitizing tumor cells to the DNA damaging effects of PUVA.

The data presented here suggests that the antitumor effects of PUVA can be mediated through DNA-independent mechanisms. It is possible that inhibition of the ErbB2 signaling axis may sensitize tumor cells to the DNA damaging effects of PUVA therapy by inhibiting P3K-Akt regulated DNA damage repair enzymes. In this context, ErbB2 targeted therapies have previously been shown to sensitize tumor cells to radiation therapy [25]. Therefore, therapeutic interventions, including PUVA alone or in combination with ErbB targeted therapies such as neratinib that can simultaneously damage DNA and also block ErbB-regulated survival pathways including those that repair damaged DNA, represent an attractive therapeutic strategy in treatment naïve ErbB2+ tumors and those that have developed resistance to ErbB2 targeted therapies through activation of alternate pathways e.g. ErbB3-EGFR-PDK1-Akt (T308) signaling axis, and express nuclear truncated ErbB2 receptors that elude the inhibition by existing ErbB2 targeted therapies.
